# The State of Music Therapy Studies in the Past 20 Years: A Bibliometric Analysis

**DOI:** 10.3389/fpsyg.2021.697726

**Published:** 2021-06-10

**Authors:** Kailimi Li, Linman Weng, Xueqiang Wang

**Affiliations:** ^1^School of Kinesiology, Shanghai University of Sport, Shanghai, China; ^2^Department of Sport Rehabilitation, Shanghai University of Sport, Shanghai, China; ^3^Department of Sport Rehabilitation Medicine, Shanghai Shangti Orthopedic Hospital, Shanghai, China

**Keywords:** music therapy, aged, bibliometrics, health, web of science

## Abstract

**Purpose:** Music therapy is increasingly being used to address physical, emotional, cognitive, and social needs of individuals. However, publications on the global trends of music therapy using bibliometric analysis are rare. The study aimed to use the CiteSpace software to provide global scientific research about music therapy from 2000 to 2019.

**Methods:** Publications between 2000 and 2019 related to music therapy were searched from the Web of Science (WoS) database. The CiteSpace V software was used to perform co-citation analysis about authors, and visualize the collaborations between countries or regions into a network map. Linear regression was applied to analyze the overall publication trend.

**Results:** In this study, a total of 1,004 studies met the inclusion criteria. These works were written by 2,531 authors from 1,219 institutions. The results revealed that music therapy publications had significant growth over time because the linear regression results revealed that the percentages had a notable increase from 2000 to 2019 (*t* = 14.621, *P* < 0.001). The United States had the largest number of published studies (362 publications), along with the following outputs: citations on WoS (5,752), citations per study (15.89), and a high H-index value (37). The three keywords “efficacy,” “health,” and “older adults,” emphasized the research trends in terms of the strongest citation bursts.

**Conclusions:** The overall trend in music therapy is positive. The findings provide useful information for music therapy researchers to identify new directions related to collaborators, popular issues, and research frontiers. The development prospects of music therapy could be expected, and future scholars could pay attention to the clinical significance of music therapy to improve the quality of life of people.

## Introduction

Music therapy is defined as the evidence-based use of music interventions to achieve the goals of clients with the help of music therapists who have completed a music therapy program (Association, 2018). In the United States, music therapists must complete 1,200 h of clinical training and pass the certification exam by the Certification Board for Music Therapists (Devlin et al., 2019). Music therapists use evidence-based music interventions to address the mental, physical, or emotional needs of an individual (Gooding and Langston, 2019). Also, music therapy is used as a solo standard treatment, as well as co-treatment with other disciplines, to address the needs in cognition, language, social integration, and psychological health and family support of an individual (Bronson et al., 2018). Additionally, music therapy has been used to improve various diseases in different research areas, such as rehabilitation, public health, clinical care, and psychology (Devlin et al., 2019). With neurorehabilitation, music therapy has been applied to increase motor activities in people with Parkinson's disease and other movement disorders (Bernatzky et al., 2004; Devlin et al., 2019). However, limited reviews about music therapy have utilized universal data and conducted massive retrospective studies using bibliometric techniques. Thus, this study demonstrates music therapy with a broad view and an in-depth analysis of the knowledge structure using bibliometric analysis of articles and publications.

Bibliometrics turns the major quantitative analytical tool that is used in conducting in-depth analyses of publications (Durieux and Gevenois, 2010; Gonzalez-Serrano et al., 2020). There are three types of bibliometric indices: (a) the quantity index is used to determine the number of relevant publications, (b) the quality index is employed to explore the characteristics of a scientific topic in terms of citations, and (c) the structural index is used to show the relationships among publications (Durieux and Gevenois, 2010; Gonzalez-Serrano et al., 2020). In this study, the three types of bibliometric indices will be applied to conduct an in-depth analysis of publications in this frontier.

While research about music therapy is extensively available worldwide, relatively limited studies use bibliometric methods to analyze the global research about this topic. The aim of this study is to use the CiteSpace software to perform a bibliometric analysis of music therapy research from 2000 to 2019. CiteSpace V is visual analytic software, which is often utilized to perform bibliometric analyses (Falagas et al., 2008; Ellegaard and Wallin, 2015). It is also a tool applied to detect trends in global scientific research. In this study, the global music therapy research includes publication outputs, distribution and collaborations between authors/countries or regions/institutions, intense issues, hot articles, common keywords, productive authors, and connections among such authors in the field. This study also provides helpful information for researchers in their endeavor to identify gaps in the existing literature.

## Materials and Methods

### Search Strategy

The data used in this study were obtained from WoS, the most trusted international citation database in the world. This database, which is run by Thomson & Reuters Corporation (Falagas et al., 2008; Durieux and Gevenois, 2010; Chen C. et al., 2012; Ellegaard and Wallin, 2015; Miao et al., 2017; Gonzalez-Serrano et al., 2020), provides high-quality journals and detailed information about publications worldwide. In this study, publications were searched from the WoS Core Collection database, which included eight indices (Gonzalez-Serrano et al., 2020). This study searched the publications from two indices, namely, the Science Citation Index Expanded and the Social Sciences Citation Index. As the most updated publications about music therapy were published in the 21st century, publications from 2000 to 2019 were chosen for this study. We performed data acquisition on July 26, 2020 using the following search terms: title = (“music therapy”) and time span = 2000–2019.

### Inclusion Criteria

**Figure 1:**
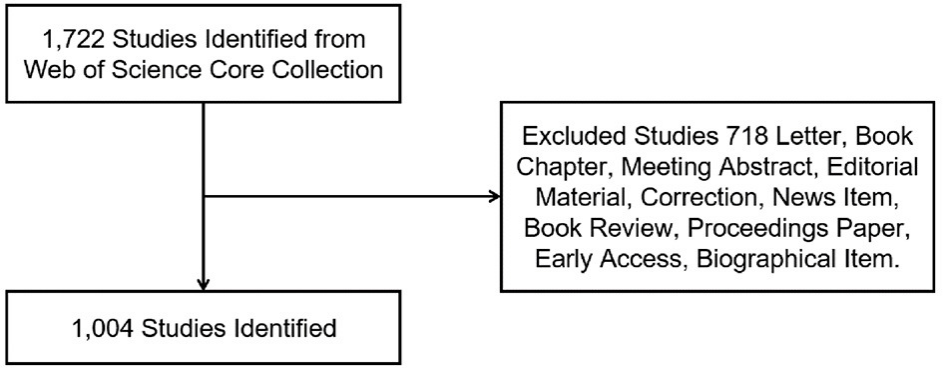
Flow chart of music therapy articles and reviews inclusion.

Figure 1 presents the inclusion criteria. The title field was music therapy (TI = music therapy), and only reviews and articles were chosen as document types in the advanced search. Other document types, such as letters, editorial materials, and book reviews, were excluded. Furthermore, there were no species limitations set. This advanced search process returned 718 articles. In the end, a total of 1,004 publications were obtained and were analyzed to obtain comprehensive perspectives on the data.

### Data Extraction

Author Lin-Man Weng extracted the publications and applied the EndNote software and Microsoft Excel 2016 to conduct analysis on the downloaded publications from the WoS database. Additionally, we extracted and recorded some information of the publications, such as citation frequency, institutions, authors' countries or regions, and journals as bibliometric indicators. The H-index is utilized as a measurement of the citation frequency of the studies for academic journals or researchers (Wang et al., 2019).

### Analysis Methods

The objective of bibliometrics can be described as the performance of studies that contributes to advancing the knowledge domain through inferences and explanations of relevant analyses (Castanha and Grácio, 2014; Merigó et al., 2019; Mulet-Forteza et al., 2021). CiteSpace V is a bibliometric software that generates information for better visualization of data. In this study, the CiteSpace V software was used to visualize six science maps about music therapy research from 2000 to 2019: the network of author co-citation, collaboration network among countries and regions, relationship of institutions interested in the field, network map of co-citation journals, network map of co-cited references, and the map (timeline view) of references with co-citation on top music therapy research. As noted, a co-citation is produced when two publications receive a citation from the same third study (Small, 1973; Merigó et al., 2019).

In addition, a science map typically features a set of points and lines to present collaborations among publications (Chen, 2006). A point is used to represent a country or region, author, institution, journal, reference, or keyword, whereas a line represents connections among them (Zheng and Wang, 2019), with stronger connections indicated by wider lines. Furthermore, the science map includes nodes, which represent the citation frequencies of certain themes. A burst node in the form of a red circle in the center indicates the number of co-occurrence or citation that increases over time. A purple node represents centrality, which indicates the significant knowledge presented by the data (Chen, 2006; Chen H. et al., 2012; Zheng and Wang, 2019). The science map represents the keywords and references with citation bursts. Occurrence bursts represent the frequency of a theme (Chen, 2006), whereas citation bursts represent the frequency of the reference. The citation bursts of keywords and references explore the trends and indicate whether the relevant authors have gained considerable attention in the field (Chen, 2006). Through this kind of map, scholars can better understand emerging trends and grasp the hot topics by burst detection analysis (Liang et al., 2017; Miao et al., 2017).

## Results

### Publication Outputs and Time Trends

**Figure 2:**
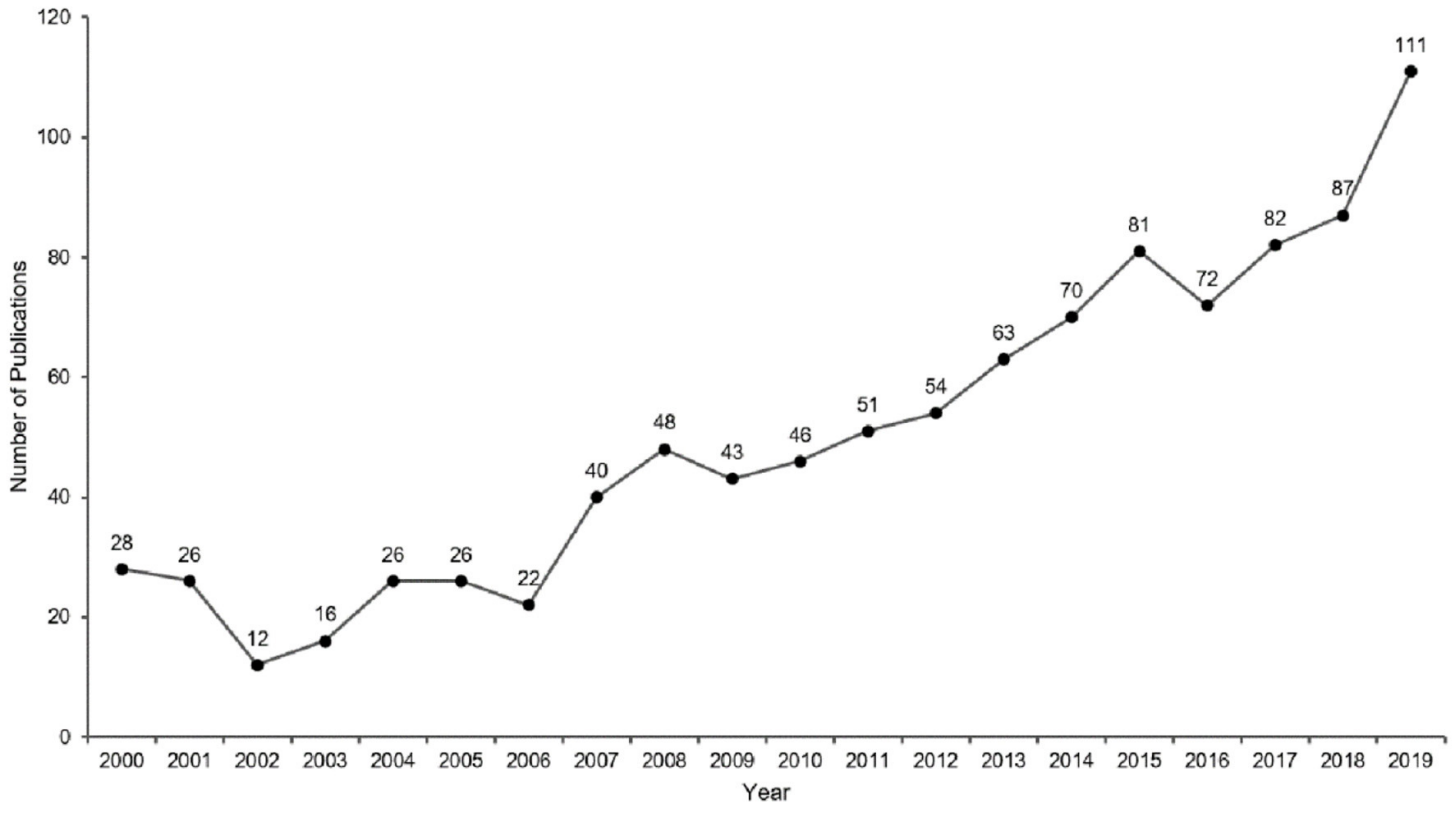
Annual publication outputs of music therapy from 2000 to 2019.

A total of 1,004 articles and reviews related to music therapy research met the criteria. The details of annual publications are presented in Figure 2. As can be seen, there were <30 annual publications between 2000 and 2006. The number of publications increased steadily between 2007 and 2015. It was 2015, which marked the first time over 80 articles or reviews were published. The significant increase in publications between 2018 and 2019 indicated that a growing number of researchers became interested in this field. Linear regression can be used to analyze the trends in publication outputs. In this study, the linear regression results revealed that the percentages had a notable increase from 2000 to 2019 (*t* = 14.621, *P* < 0.001). Moreover, the *P* < 0.05, indicating statistical significance. Overall, the publication outputs increased from 2000 to 2019.

### Distribution by Country or Region and Institution

**Figure 3:**
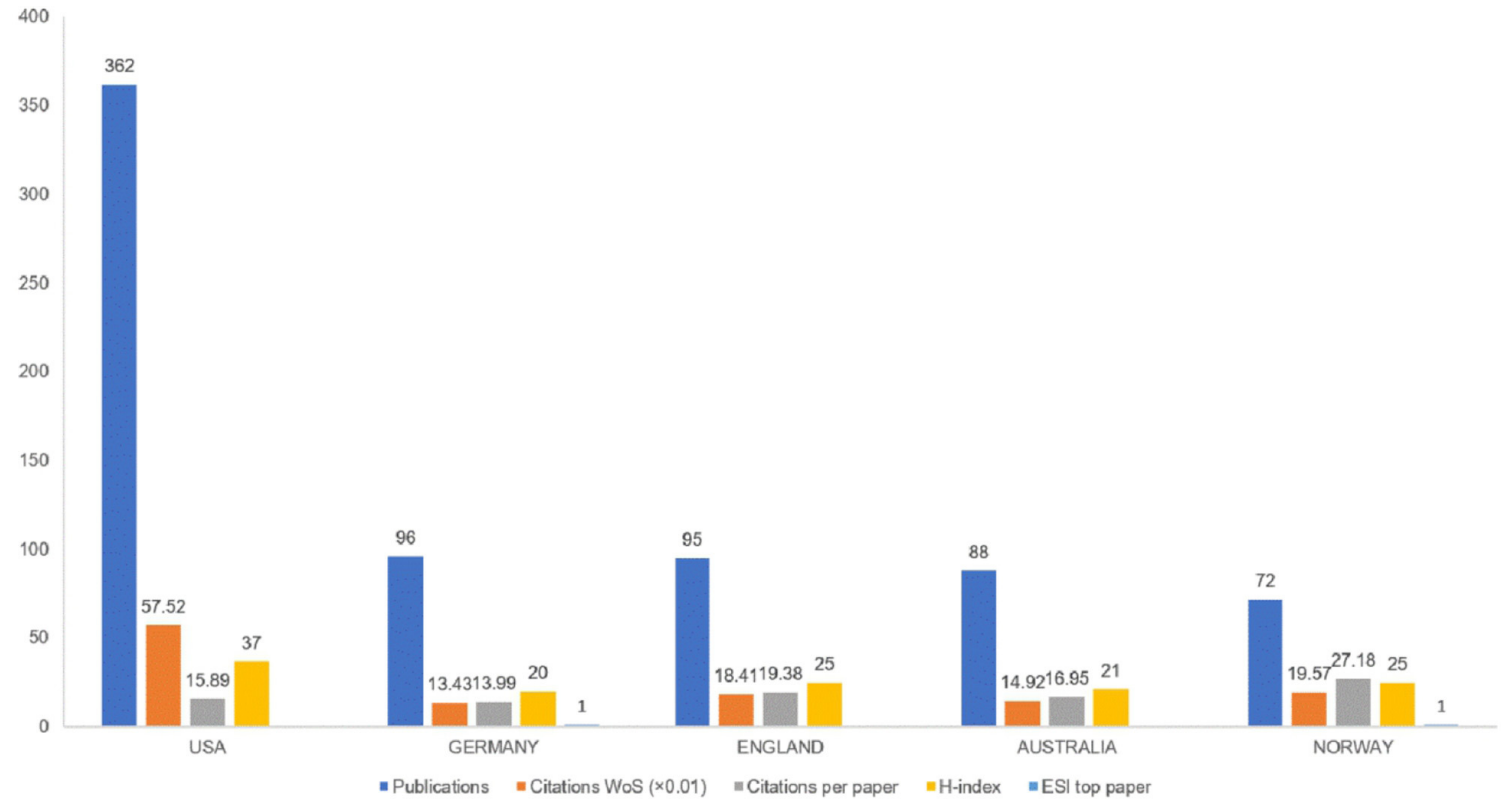
Publications, citations on WoS (×0.01), citations per study, H-index, and ESL top study among top five countries or regions.

**Figure 4:**
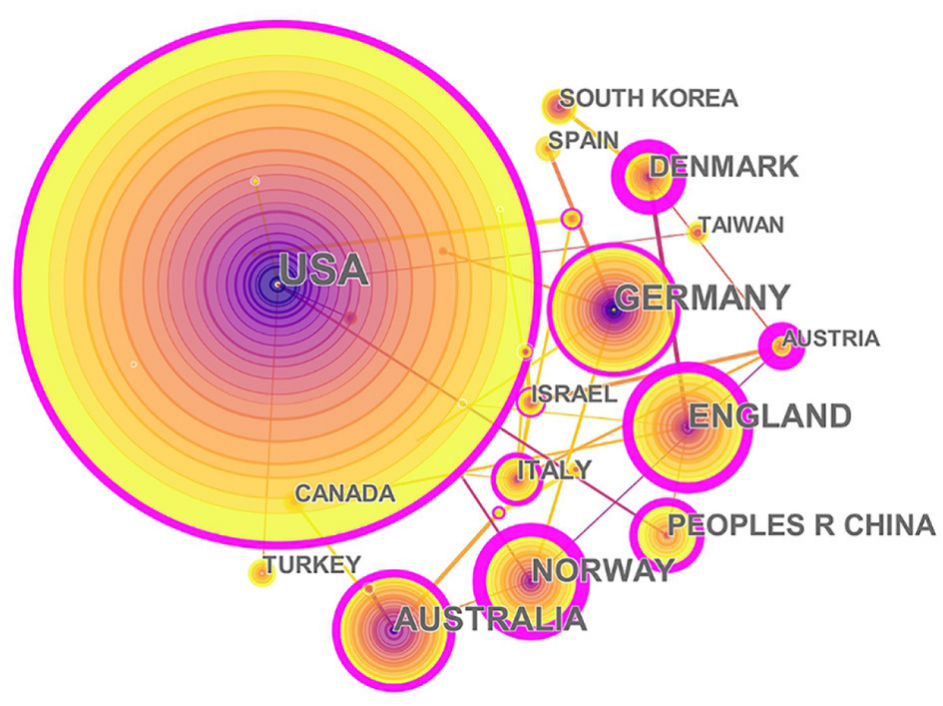
The collaborations of countries or regions interested in the field. In this map, the node represents a country, and the link represents the cooperation relationship between two countries. A larger node represents more publications in the country. A thicker purple circle represents greater influence in this field.

**Figure 5:**
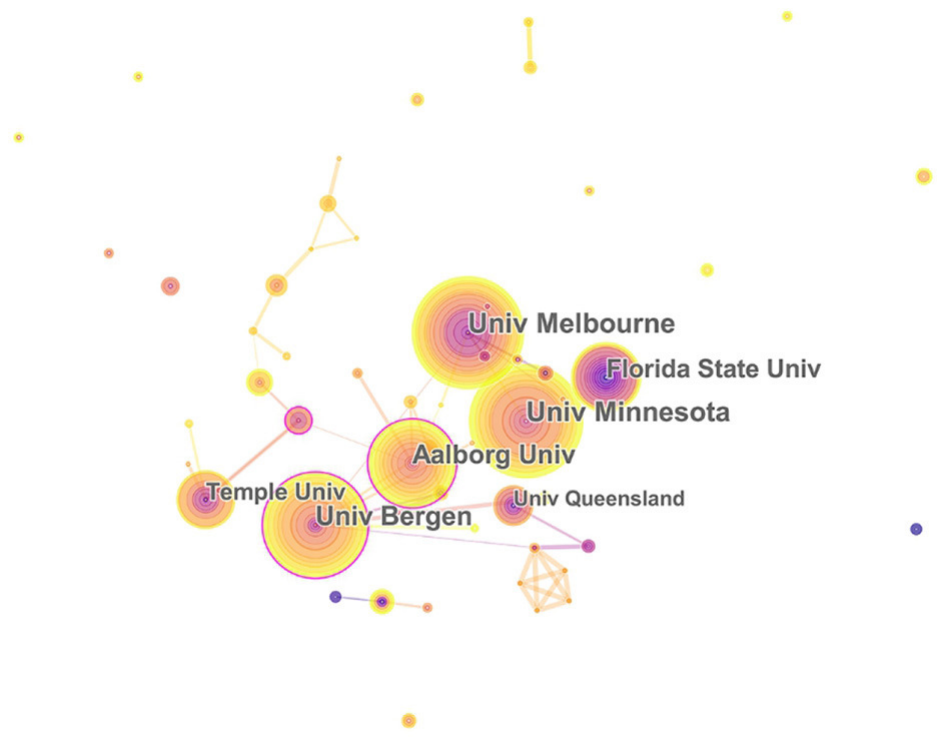
The relationship of institutions interested in the field. University of Melbourne, Florida State University, University of Minnesota, Aalborg University, Temple University, University of Queensland, and University of Bergen. In this map, the node represents an institution, and the link represents the cooperation relationship between two institutions. A larger node represents more publications in the institution. A thicker purple circle represents greater influence in this field.

The 1,004 articles and reviews collected were published in 49 countries and regions. Table 1 presents the top 10 countries or regions. Figure 3 shows an intuitive comparison of the citations on WoS, citations per study, Hirsch index (H-index), and major essential science indicator (ESI) studies of the top five countries or regions. The H-index is a kind of index that is applied in measuring the wide impact of the scientific achievements of authors. The United States had the largest number of published studies (362 publications), along with the following outputs: citations on WoS (5,752), citations per study (15.89), and a high H-index value (37). Norway has the largest number of citations per study (27.18 citations). Figure 4 presents the collaboration networks among countries or regions. The collaboration network map contained 32 nodes and 38 links. The largest node can be found in the United States, which meant that the United States had the largest number of publications in the field. Meanwhile, the deepest purple circle was located in Austria, which meant that Austria is the country with the most number of collaborations with other countries or regions in this research field. A total of 1,219 institutions contributed various music therapy-related publications. Figure 5 presents the collaborations among institutions. As can be seen, the University of Melbourne is the most productive institution in terms of the number of publications (45), followed by the University of Minnesota (43), and the University of Bergen (39). The top 10 institutions featured in Table 2 contributed 28.884% of the total articles and reviews published. Among these, Aalborg University had the largest centrality (0.13). The top 10 productive institutions with details are shown in Table 2.

**Table 1:** Top 10 countries or regions of origin of study in the music therapy research field.

**Rank**	**Country or regions**	**Publications**	**Percentage (%)**	**Citations WoS**	**Citations per paper**	**H-index**
1	USA	362	36.056	5,752	15.89	37
2	Germany	96	9.562	1,343	13.99	20
3	England	95	9.462	1,841	19.38	25
4	Australia	88	8.765	1,492	16.95	21
5	Norway	72	7.171	1,957	27.18	25
6	China	53	5.279	767	14.47	17
7	Denmark	45	4.482	1,218	27.07	17
8	Italy	39	3.884	987	25.31	14
9	Canada	30	2.988	401	13.37	10
10	Israel	29	2.888	346	11.93	9

**Table 2:** Top 10 institutions that contributed to publications in the music therapy field.

**Rank**	**Institution**	**Publications**	**Percentage (%)**	**Centrality**
1	University of Melbourne	45	4.482	0.08
2	University of Minnesota	43	4.283	0.00
3	University of Bergen	39	3.884	0.12
4	Florida State University	33	3.287	0.00
5	Aalborg University	32	3.187	0.13
6	Temple University	27	2.689	0.04
7	University of Kansas	20	1.992	0.00
8	University of Queensland	20	1.992	0.00
9	Anglia Ruskin University	16	1.594	0.08
10	Bar Ilan University	15	1.494	0.00

### Distribution by Journals

**Figure 6:**
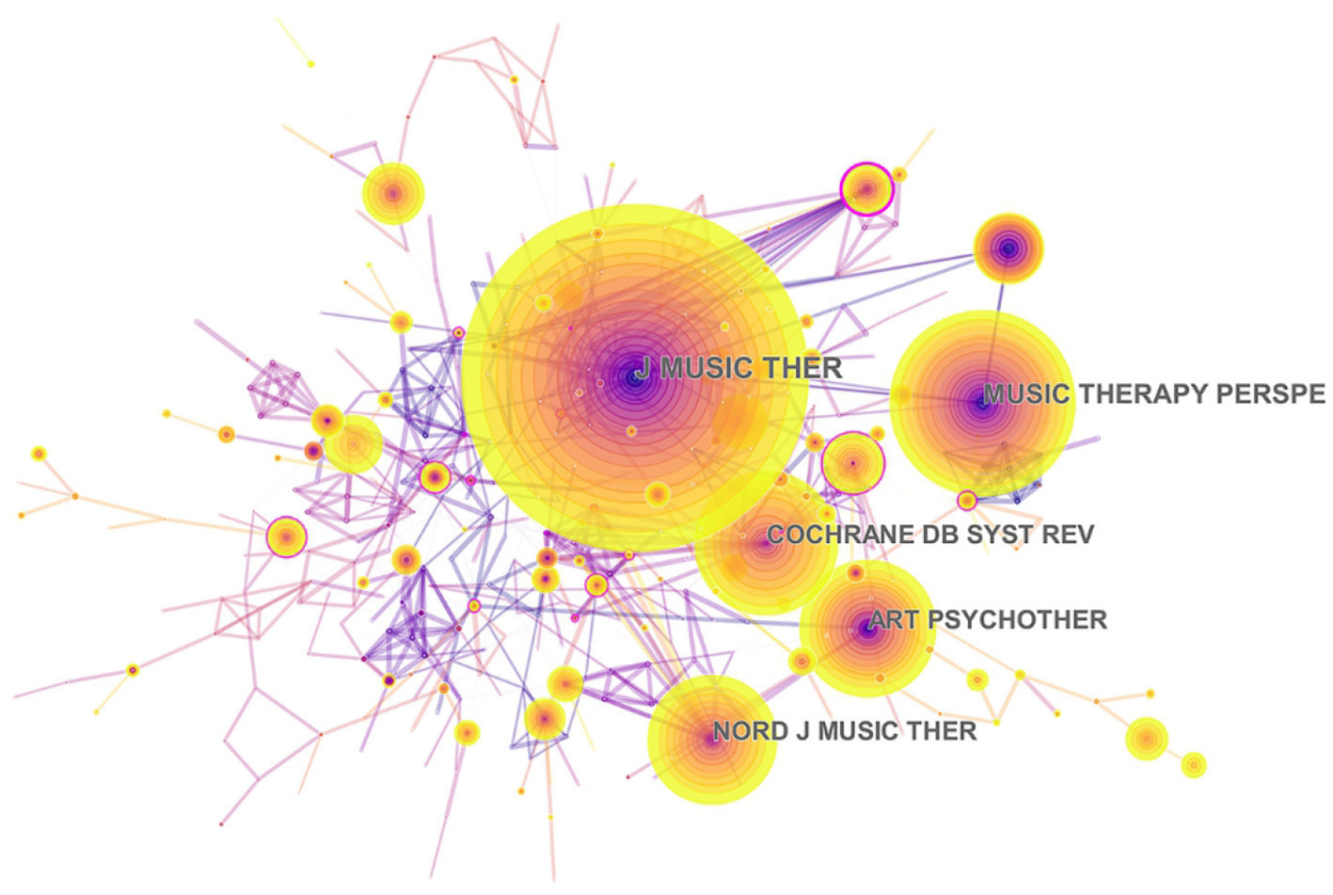
Network map of co-citation journals engaged in music therapy from 2000 to 2019. Journal of Music Therapy, Arts in Psychotherapy, Nordic Journal of Music Therapy, Music Therapy Perspectives, Cochrane Database of Systematic Reviews. In this map, the node represents a journal, and the link represents the co-citation frequency between two journals. A larger node represents more publications in the journal. A thicker purple circle represents greater influence in this field.

Table 3 presents the top 10 journals that published articles or reviews in the music therapy field. The publications are mostly published in these journal fields, such as Therapy, Medical, Psychology, Neuroscience, Health and Clinical Care. The impact factors (IF) of these journals ranged between 0.913 and 7.89 (average IF: 2.568). Four journals had an impact factor >2, of which Cochrane Database of Systematic Reviews had the highest IF, 2019 = 7.89. In addition, the Journal of Music Therapy (IF: 2019 = 1.206) published 177 articles or reviews (17.629%) about music therapy in the past two decades, followed by the Nordic Journal of Music Therapy (121 publications, 12.052%, IF: 2019 = 0.913), and Arts in Psychotherapy (104 publications, 10.359%, IF: 2019 = 1.322). Furthermore, the map of the co-citation journal contained 393 nodes and 759 links (Figure 6). The high co-citation count identifies the journals with the greatest academic influence and key positions in the field. The Journal of Music Therapy had the maximum co-citation counts (658), followed by Cochrane Database of Systematic Reviews (281), and Arts in Psychotherapy (279). Therefore, according to the analysis of the publications and co-citation counts, the Journal of Music Therapy and Arts in Psychotherapy occupied key positions in this research field.

**Table 3:** Top 10 journals that published articles in the music therapy field.

**Rank**	**Journal**	**Publications**	**Percentage (%)**	**IF** ** (2019)**
1	Journal of Music Therapy	177	17.629	1.206
2	Nordic Journal of Music Therapy	121	12.052	0.913
3	Arts in Psychotherapy	104	10.359	1.322
4	Analysis of the New York Academy of Sciences	18	1.793	4.728
5	Complementary Therapies in Medicine	12	1.195	2.063
6	Journal of Clinical Nursing	10	0.996	1.972
7	Journal of Palliative care	10	0.996	1.200
8	Cochrane Database of Systematic Reviews	9	0.896	7.890
9	Frontiers in Human Neuroscience	9	0.896	2.673
10	Psychology of Music	9	0.896	1.712

*IF, impact factor*.

### Distribution by Authors

**Figure 7:**
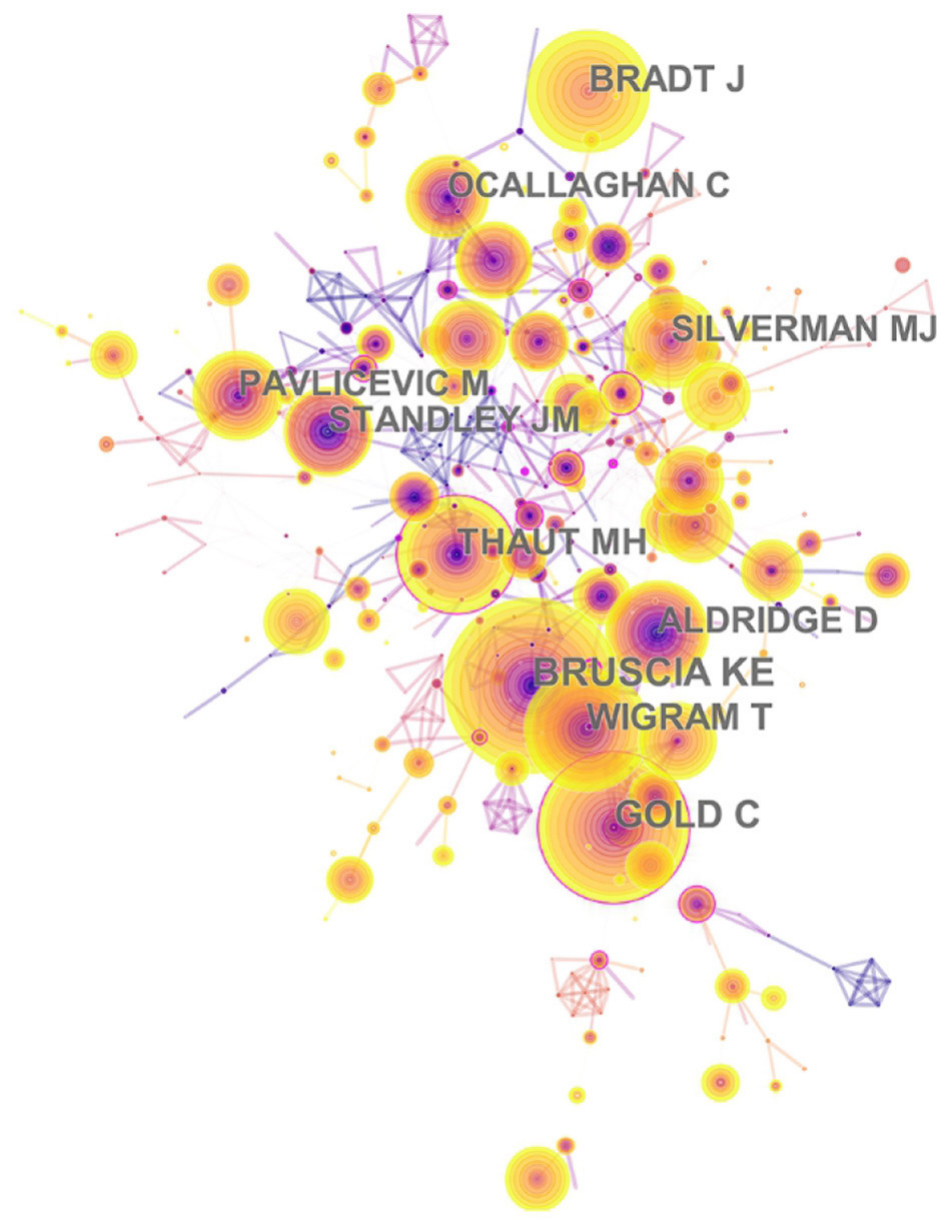
The network of author co-citaion. In this map, the node represents an author, and the link represents the co-citation frequency between two authors. A larger node represents more publications of the author. A thicker purple circle represents greater influence in this field.

A total of 2,531 authors contributed to the research outputs related to music therapy. Author Silverman MJ published most of the studies (46) in terms of number of publications, followed by Gold C (41), Magee WL (19), O'Callaghan C (15), and Raglio A (15). According to co-citation counts, Bruscia KE (171 citations) was the most co-cited author, followed by Gold C (147 citations), Wigram T (121 citations), and Bradt J (117 citations), as presented in Table 4. In Figure 7, these nodes highlight the co-citation networks of the authors. The large-sized node represented author Bruscia KE, indicating that this author owned the most co-citations. Furthermore, the linear regression results revealed a remarkable increase in the percentages of multiple articles of authors (*t* = 13.089, *P* < 0.001). These also indicated that cooperation among authors had increased remarkably, which can be considered an important development in music therapy research.

**Table 4:** Top five authors of publications and top five authors of co-citation counts.

**Rank**	**Author**	**Publications**	**Percentage (%)**	**Centrality**	**Cited author**	**Co-citation counts**
1	Silverman MJ	46	4.582	0.00	Bruscia KE	171
2	Gold C	41	4.084	0.06	Gold C	147
3	Magee WL	19	1.892	0.01	Wigram T	121
4	O'Callaghan C	15	1.494	0.01	Bradt J	117
5	Raglio A	15	1.494	0.00	Thaut MH	116

### Analysis of Keywords

**Figure 8:**
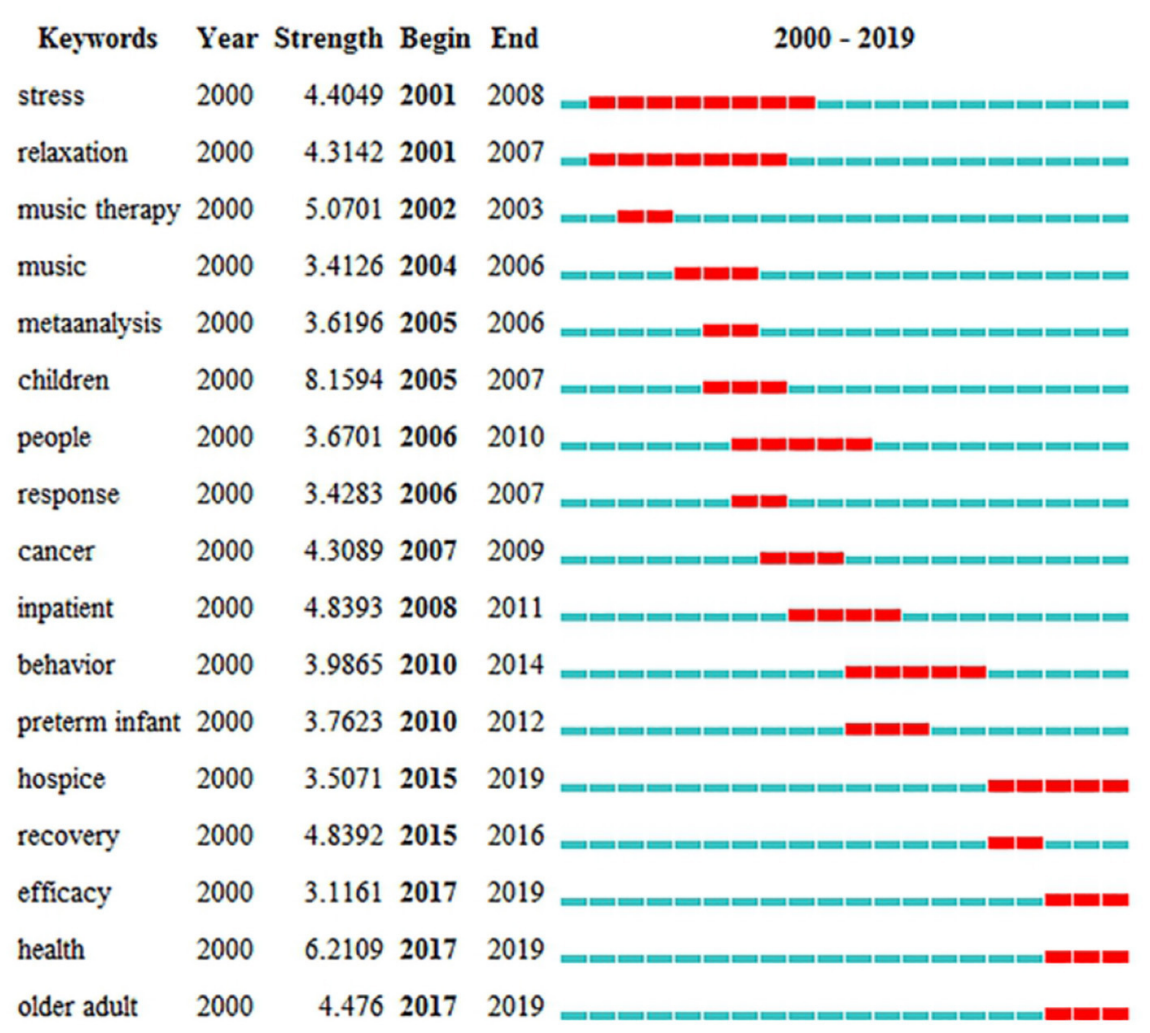
The strongest citation bursts of the top 17 keywords. The red measures indicate frequent citation of keywords, and the green measures indicate infrequent citation of keywords.

The results of keywords analysis indicated research hotspots and help scholars identify future research topics. Table 5 highlights 20 keywords with the most frequencies, such as “music therapy,” “anxiety,” “intervention,” “children,” and “depression.” The keyword “autism” has the highest centrality (0.42). Figure 8 shows the top 17 keywords with the strongest citation bursts. By the end of 2019, keyword bursts were led by “hospice,” which had the strongest burst (3.5071), followed by “efficacy” (3.1161), “health” (6.2109), and “older adult” (4.476).

**Table 5:** Top 20 keywords with the most frequency and centrality in music therapy study.

**Rank**	**Keyword**	**Frequency**	**Keyword**	**Centrality**
1	Music therapy	486	Autism	0.42
2	Anxiety	149	People	0.34
3	Intervention	116	Brain	0.32
4	Children	94	Schizophrenia	0.23
5	Depression	90	Quality of life	0.21
6	Pain	76	Perception	0.19
7	Dementia	71	Plasticity	0.17
8	Music	62	Parent	0.15
9	Randomized controlled trial	57	Adolescent	0.14
10	Quality of life	50	Behavior	0.12
11	People	48	Mental health	0.12
12	Relaxation	48	Response	0.12
13	Recovery	45	Recovery	0.11
14	Stress	45	Stress	0.11
15	Care	45	Care	0.10
16	Cancer	45	Preterm infant	0.10
17	Behavior	42	Dementia	0.09
18	Symptom	40	Reliability	0.09
19	Rehabilitation	39	Mother	0.09
20	Adolescent	38	Self esteem	0.09

### Analysis of Co-cited References

**Figure 9:**
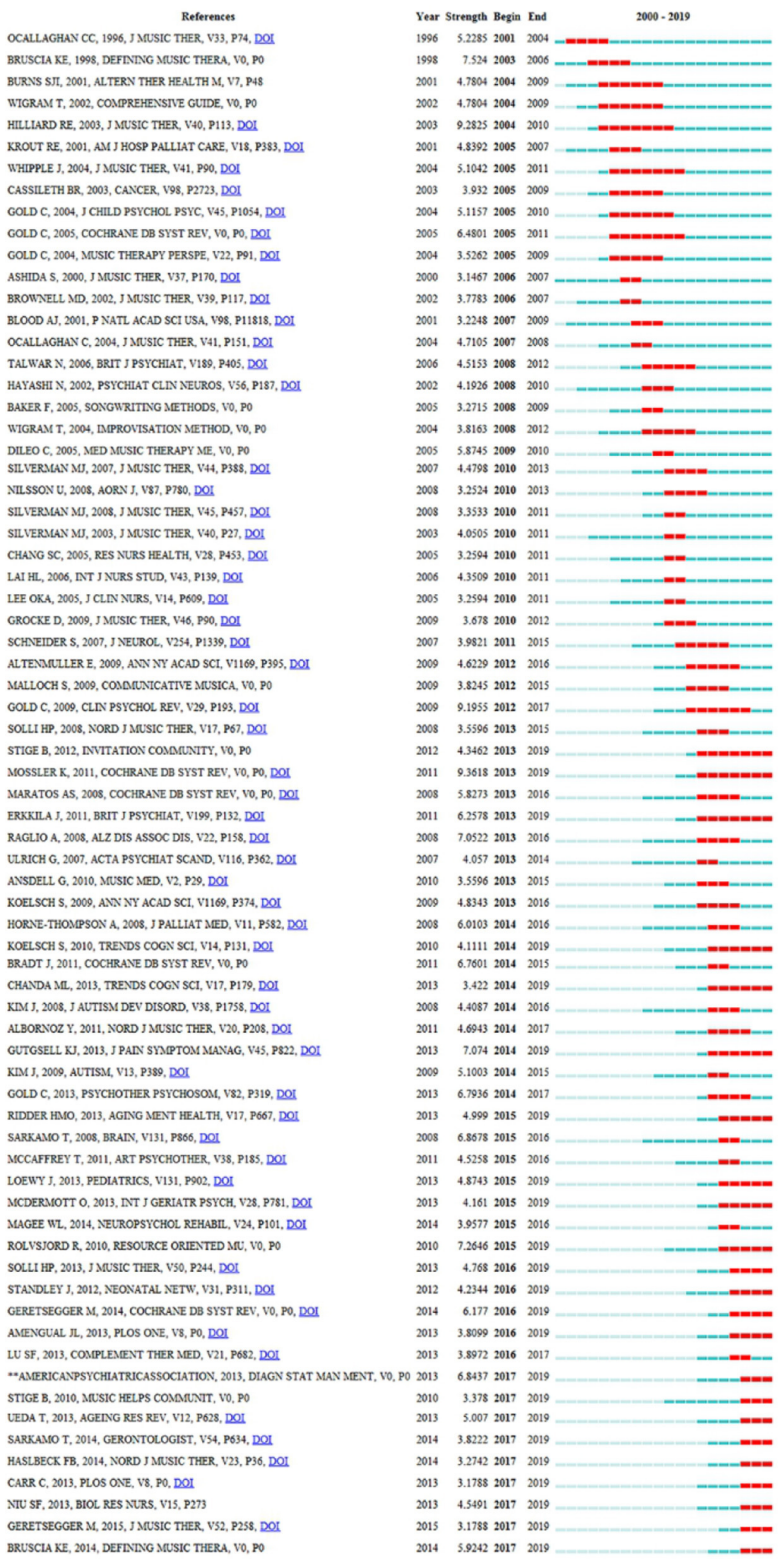
The strongest citation bursts among the top 71 references. The red measures indicate frequent citation of studies, and the green measures indicate infrequent citation of studies.

The analysis of co-cited references is a significant indicator in the bibliometric method (Chen, 2006). The top five co-cited references and their main findings are listed in Table 6. These are regarded as fundamental studies for the music therapy knowledge base. In terms of co-citation counts, “individual music therapy for depression: randomized controlled trial” was the key reference because it had the most co-citation counts. This study concludes that music therapy mixed with standard care is an effective way to treat working-age people with depression. The authors also explained that music therapy is a valuable enhancement to established treatment practices (Erkkilä et al., 2011). Meanwhile, the strongest citation burst of reference is regarded as the main knowledge of the trend (Fitzpatrick, 2005). Figure 9 highlights the top 71 strongest citation bursts of references from 2000 to 2019. As can be seen, by the end of 2019, the reference burst was led by author Stige B, and the strongest burst was 4.3462.

**Table 6:** Top five co-cited references with co-citation counts in the study of music therapy from 2000 to 2019.

**Rank**	**Cited reference**	**Co-citation counts**	**Publication year**	**Main findings**
1	Individual music therapy for depression: randomized controlled trial	43	2011	Music therapy with its specific qualities is a valuable enhancement to working-age people with depression.
2	Dose-response relationship in music therapy for people with serious mental disorders: systematic review and meta-analysis	39	2009	Music therapy is an effective treatment which helps people with psychotic and non-psychotic mental disorders.
3	Music therapy for people with schizophrenia and schizophrenia-like disorders	32	2011	Music therapy can help people improve their emotional and relational competencies.
4	Music therapy for depression	29	2008	Music therapy is accepted by people with depression and is associated with improvements in mood.
5	Resource-oriented music therapy in mental health care	29	2010	An introduction to the resource-oriented approach to music therapyin mental health care.

**Figure 10:**
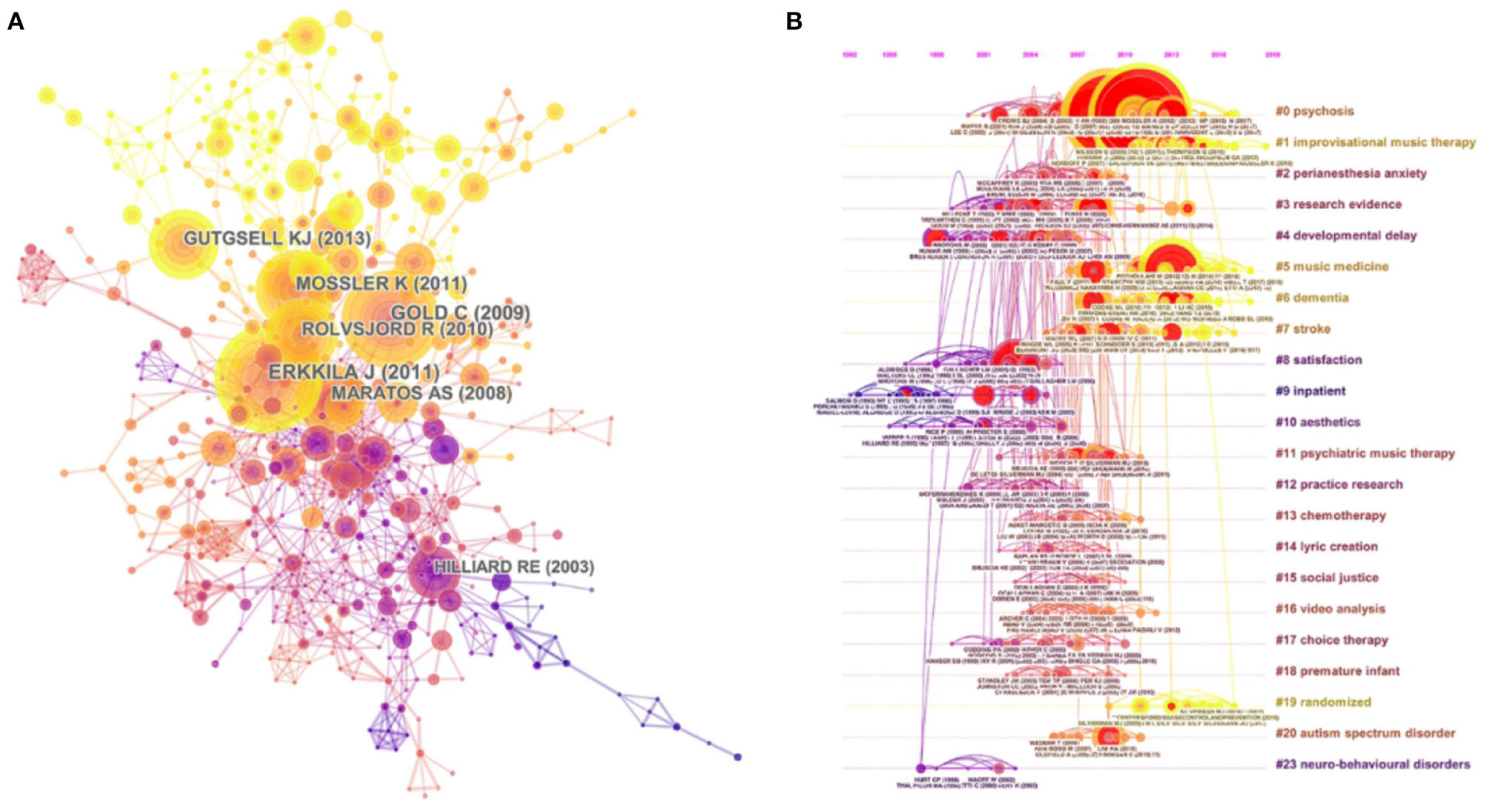
**(A)** The network map of co-cited references and **(B)** the map (timeline view) of references with co-citation on top music therapy research. In these maps, the node represents a study, and the link represents the co-citation frequency between two studies. A larger node represents more publications of the author. A thicker purple circle represents greater influence in this field. **(A)** The nodes in the same color belong to the same cluster. **(B)** The nodes on the same line belong to the same cluster.

Figure 10A presents the co-cited reference map containing 577 nodes and 1,331 links. The figure explains the empirical relevance of a considerable number of articles and reviews. Figure 10B presents the co-citation map (timeline view) of reference from publications on top music therapy research. The timeline view of clusters shows the research progress of music therapy in a particular period of time and the thematic concentration of each cluster. “Psychosis” was labeled as the largest cluster (#0), followed by “improvisational music therapy” (#1) and “paranesthesia anxiety” (#2). These clusters have also remained hot topics in recent years. Furthermore, the result of the modularity Q score was 0.8258. That this value exceeded 0.5 indicated that the definitions of the subdomain and characters of clusters were distinct. In addition, the mean silhouette was 0.5802, which also exceeded 0.5. The high homogeneity of individual clusters indicated high concentration in different research areas.

## Discussion

### Global Trends in Music Therapy Research

This study conducted a bibliometric analysis of music therapy research from the past two decades. The results, which reveal that music therapy studies have been conducted throughout the world, among others, can provide further research suggestions to scholars. In terms of the general analysis of the publications, the features of published articles and reviews, prolific countries or regions, and productive institutions are summarized below.

I. The distribution of publication year has been increasing in the past two decades. The annual publication outputs of music therapy from 2000 to 2019 were divided into three stages: beginning, second, and third. In the beginning stage, there were <30 annual publications from 2000 to 2006. The second stage was between 2007 and 2014. The number of publications increased steadily. It was 2007, which marked the first time 40 articles or reviews were published. The third stage was between 2015 and 2019. The year 2015 was the key turning point because it was the first time 80 articles or reviews were published. The number of publications showed a downward trend in 2016 (72), but it was still higher than the average number of the previous years. Overall, music therapy-related research has received increasing attention among scholars from 2000 to 2020.

II. The articles and reviews covered about 49 countries or regions, and the prolific countries or regions were mainly located in the North American and European continents. According to citations on WoS, citations per study, and the H-index, music therapy publications from developed countries, such as United States and Norway, have greater influence than those from other countries. In addition, China, as a model of a developing country, had published 53 studies and ranked top six among productive countries.

III. In terms of the collaboration map of institutions, the most productive universities engaged in music therapy were located in the United States, namely, University of Minnesota (43 publications), Florida State University (33 publications), Temple University (27 publications), and University of Kansas (20 publications). It indicated that institutions in the US have significant impacts in this area.

**Figure 11:**
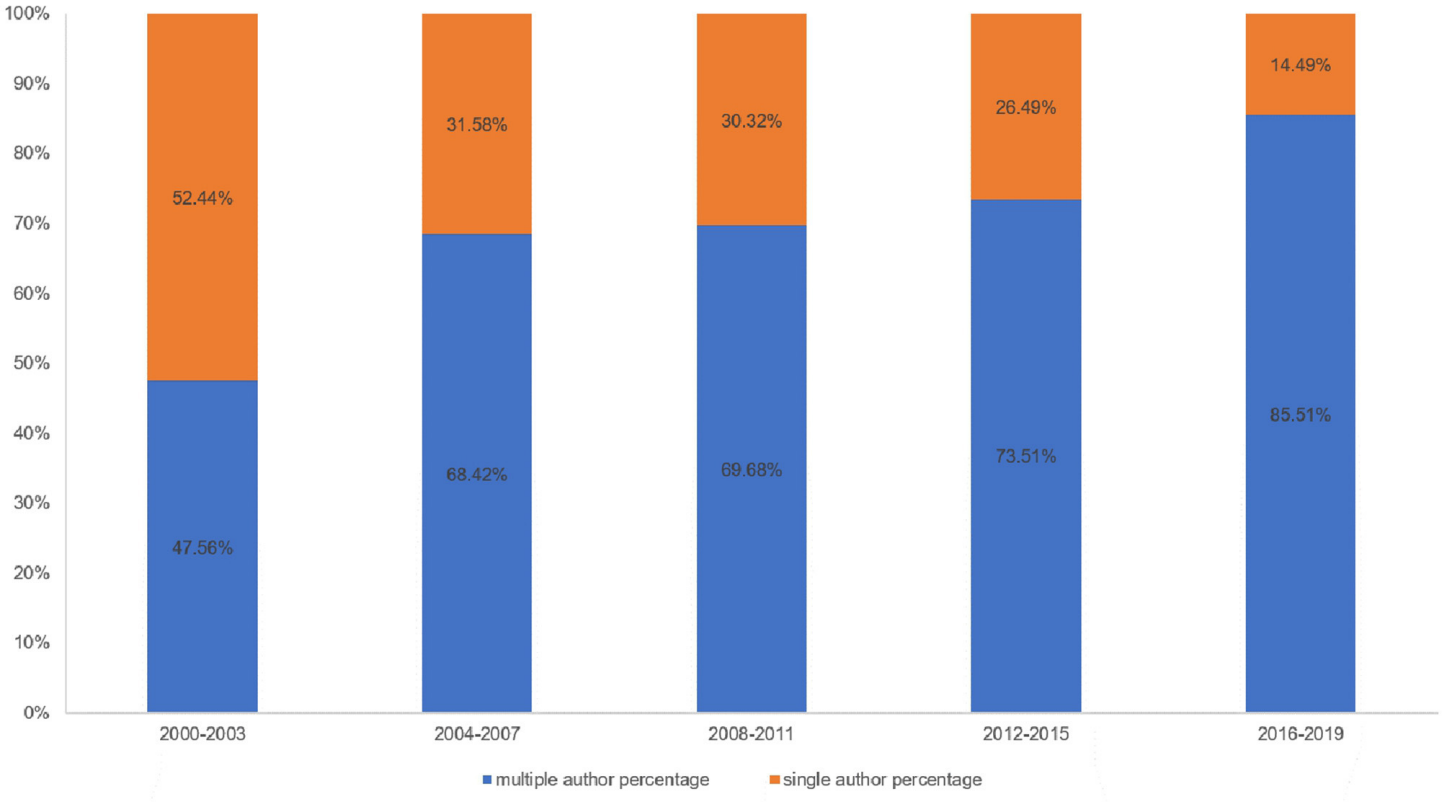
The percentage of single- vs. multiple-authored articles. Blue bars mean multiple-author percentage; orange bars mean single-author percentage.

IV. According to author co-citation counts, scholars can focus on the publications of such authors as Bruscia KE, Gold C, and Wigram T. These three authors come from the United States, Norway, and Denmark, and it also reflected that these three countries are leading the research trend. Author Bruscia KE has the largest co-citation counts and is based at Temple University. He published many music therapy studies about assessment and clinical evaluation in music therapy, music therapy theories, and therapist experiences. These publications laid a foundation and facilitate the development of music therapy. In addition, in Figure 11, the multi-authored articles between 2000 and 2003 comprised 47.56% of the sample, whereas the publications of multi-authored articles increased significantly from 2016 to 2019 (85.51%). These indicated that cooperation is an effective factor in improving the quality of publications.

### Research Focus on the Research Frontier and Hot Topics

According to the science map analysis, hot music therapy topics among publications are discussed.

I. The cluster “#1 improvisational music therapy” (IMT) is the current research frontier in the music therapy research field. In general, music therapy has a long research tradition within autism spectrum disorders (ASD), and there have been more rigorous studies about it in recent years. IMT for children with autism is described as a child-centered method. Improvisational music-making may enhance social interaction and expression of emotions among children with autism, such as responding to communication acts (Geretsegger et al., 2012, 2015). In addition, IMT is an evidence-based treatment approach that may be helpful for people who abuse drugs or have cancer. A study applied improving as a primary music therapeutic practice, and the result indicated that IMT will be effective in treating depression accompanied by drug abuse among adults (Albornoz, 2011). By applying the interpretative phenomenological analysis and psychological perspectives, a study explained the significant role of music therapy as an innovative psychological intervention in cancer care settings (Pothoulaki et al., 2012). IMT may serve as an effective additional method for treating psychiatric disorders in the short and medium term, but it may need more studies to identify the long-term effects in clinical practice.

II. Based on the analysis of co-citation counts, the top three references all applied music therapy to improve the quality of life of clients. They highlight the fact that music therapy is an effective method that can cover a range of clinical skills, thus helping people with psychological disorders, chronic illnesses, and pain management issues. Furthermore, music therapy mixed with standard care can help individuals with schizophrenia improve their global state, mental state (including negative and general symptoms), social functioning, and quality of life (Gold et al., 2009; Erkkilä et al., 2011; Geretsegger et al., 2017).

III. By understanding the keywords with the strongest citation bursts, the research frontier can be predicted. Three keywords, “efficacy,” “health,” and “older adults,” emphasized the research trends in terms of the strongest citation bursts.

- Efficacy: This refers to measuring the effectiveness of music therapy in terms of clinical skills. Studies have found that a wide variety of psychological disorders can be effectively treated with music. In the study of Fukui, patients with Alzheimer's disease listened to music and verbally communicated with their music therapist. The results showed that problematic behaviors of the patients with Alzheimer's disease decreased (Fukui et al., 2012). The aim of the study of Erkkila was to determine the efficacy of music therapy when added to standard care. The result of this study also indicated that music therapy had specific qualities for non-verbal expression and communication when patients cannot verbally describe their inner experiences (Erkkilä et al., 2011). Additionally, as summarized by Ueda, music therapy reduced anxiety and depression in patients with dementia. However, his study cannot clarify what kinds of music therapy or patients have effectiveness. Thus, future studies should investigate music therapy with good methodology and evaluation methods (Ueda et al., 2013).
- Health: Music therapy is a methodical intervention in clinical practice because it uses music experiences and relationships to promote health for adults and children (Bruscia, 1998). Also, music therapy is an effective means of achieving the optimal health and well-being of individuals and communities, because it can be individualized or done as a group activity. The stimulation from music therapy can lead to conversations, recollection of memories, and expression. The study of Gold indicated that solo music therapy in routine practice is an effective addition to usual care for mental health care patients with low motivation (Gold et al., 2013). Porter summarized that music therapy contributes to improvement for both kids and teenagers with mental health conditions, such as depression and anxiety, and increases self-esteem in the short term (Porter et al., 2017).
- Older adults: This refers to the use of music therapy as a treatment to maintain and slow down the symptoms observed in older adults (Mammarella et al., 2007; Deason et al., 2012). In terms of keywords with the strongest citation bursts, the most popular subjects of music therapy-related articles and reviews focused on children from 2005 to 2007. However, various researchers concentrated on older adults from 2017 to 2019. Music therapy was the treatment of choice for older adults with depression, Parkinson's disease, and Alzheimer's disorders (Brotons and Koger, 2000; Bernatzky et al., 2004; Johnson et al., 2011; Deason et al., 2012; McDermott et al., 2013; Sakamoto et al., 2013; Benoit et al., 2014; Pohl et al., 2020). In the study of Zhao, music therapy had positive effects on the reduction of depressive symptoms for older adults when added to standard therapies. These standard therapies could be standard care, standard drug treatment, standard rehabilitation, and health education (Zhao et al., 2016). The study of Shimizu demonstrated that multitask movement music therapy was an effective intervention to enhance neural activation in older adults with mild cognitive impairment (Shimizu et al., 2018). However, the findings of the study of Li explained that short-term music therapy intervention cannot improve the cognitive function of older adults. He also recommended that future researchers can apply a quality methodology with a long-term research design for the care needs of older adults (Li et al., 2015).

### Strengths and Limitations

To the best of our knowledge, this study was the first one to analyze large-scale data of music therapy publications from the past two decades through CiteSpace V. CiteSpace could detect more comprehensive results than simply reviewing articles and studies. In addition, the bibliometric method helped us to identify the emerging trend and collaboration among authors, institutions, and countries or regions.

This study is not without limitations. First, only articles and reviews published in the WoS Science Citation Index Expanded and Social Sciences Citation Index were analyzed. Future reviews could consider other databases, such as PubMed and Scopus. The document type labeled by publishers is not always accurate. For example, some publications labeled by WoS were not actually reviews (Harzing, 2013; Yeung, 2021). Second, the limitation may induce bias in frequency of reference. For example, some potential articles were published recently, and these studies could be not cited with frequent times. Also, in terms of obliteration by incorporation, some common knowledge or opinions become accepted that their contributors or authors are no longer cited (Merton, 1965; Yeung, 2021). Third, this review applied the quantitative analysis approach, and only limited qualitative analysis was performed in this study. In addition, we applied the CitesSpace software to conduct this bibliometric study, but the CiteSpace software did not allow us to complicate information under both full counting and fractional counting systems. Thus, future scholars can analyze the development of music therapy in some specific journals using both quantitative and qualitative indicators.

## Conclusions

This bibliometric study provides information regarding emerging trends in music therapy publications from 2000 to 2019. First, this study presents several theoretical implications related to publications that may assist future researchers to advance their research field. The results reveal that annual publications in music therapy research have significantly increased in the last two decades, and the overall trend in publications increased from 28 publications in 2000 to 111 publications in 2019. This analysis also furthers the comprehensive understanding of the global research structure in the field. Also, we have stated a high level of collaboration between different countries or regions and authors in the music therapy research. This collaboration has extremely expanded the knowledge of music therapy. Thus, future music therapy professionals can benefit from the most specialized research.

Second, this research represents several practical implications. IMT is the current research frontier in the field. IMT usually serves as an effective music therapy method for the health of people in clinical practice. Identifying the emerging trends in this field will help researchers prepare their studies on recent research issues (Mulet-Forteza et al., 2021). Likewise, it also indicates future studies to address these issues and update the existing literature. In terms of the strongest citation bursts, the three keywords, “efficacy,” “health,” and “older adults,” highlight the fact that music therapy is an effective invention, and it can benefit the health of people. The development prospects of music therapy could be expected, and future scholars could pay attention to the clinical significance of music therapy to the health of people.

Finally, multiple researchers have indicated several health benefits of music therapy, and the music therapy mechanism perspective is necessary for future research to advance the field. Also, music therapy can benefit a wide range of individuals, such as those with autism spectrum, traumatic brain injury, or some physical disorders. Future researchers can develop music therapy standards to measure clinical practice.

## Author Contributions

KL and LW: conceptualization, methodology, formal analysis, investigation, resources, writing—review, and editing. LW: software and data curation. KL: validation and writing—original draft preparation. XW: visualization, supervision, project administration, and funding acquisition. All authors contributed to the article and approved the submitted version.

## Conflict of Interest

The authors declare that the research was conducted in the absence of any commercial or financial relationships that could be construed as a potential conflict of interest.

## Abbreviations

WoS: Web of Science
ESI: essential science indicators
IF: impact factor
IMT: improvisational music therapy
ASD: autism spectrum disorder.
